# FH535, a β-catenin pathway inhibitor, represses pancreatic cancer xenograft growth and angiogenesis

**DOI:** 10.18632/oncotarget.9975

**Published:** 2016-06-13

**Authors:** Lu Liu, Qiaoming Zhi, Meng Shen, Fei-Ran Gong, Binhua P. Zhou, Lian Lian, Bairong Shen, Kai Chen, Weiming Duan, Meng-Yao Wu, Min Tao, Wei Li

**Affiliations:** ^1^ Department of Oncology, the First Affiliated Hospital of Soochow University, Suzhou, China; ^2^ Department of General Surgery, the First Affiliated Hospital of Soochow University, Suzhou, China; ^3^ Department of Hematology, the First Affiliated Hospital of Soochow University, Suzhou, China; ^4^ Markey Cancer Center, University of Kentucky, Lexington, KY, USA; ^5^ Departments of Molecular and Cellular Biochemistry, University of Kentucky College of Medicine, Lexington, KY, USA; ^6^ Department of Oncology, Suzhou Xiangcheng People's Hospital, Suzhou, China; ^7^ Department of Pathology, Suzhou Xiangcheng People's Hospital, Suzhou, China; ^8^ Center for Systems Biology, Soochow University, Suzhou, China; ^9^ PREMED Key Laboratory for Precision Medicine, Soochow University, Suzhou, China; ^10^ Jiangsu Institute of Clinical Immunology, Suzhou, China; ^11^ Institute of Medical Biotechnology, Soochow University, Suzhou, China

**Keywords:** pancreatic cancer, FH535, β-catenin, cancer stem cell, angiogenesis

## Abstract

The WNT/β-catenin pathway plays an important role in pancreatic cancer carcinogenesis. We evaluated the correlation between aberrant β-catenin pathway activation and the prognosis pancreatic cancer, and the potential of applying the β-catenin pathway inhibitor FH535 to pancreatic cancer treatment. Meta-analysis and immunohistochemistry showed that abnormal β-catenin pathway activation was associated with unfavorable outcome. FH535 repressed pancreatic cancer xenograft growth *in vivo*. Gene Ontology (GO) analysis of microarray data indicated that target genes responding to FH535 participated in stemness maintenance. Real-time PCR and flow cytometry confirmed that FH535 downregulated CD24 and CD44, pancreatic cancer stem cell (CSC) markers, suggesting FH535 impairs pancreatic CSC stemness. GO analysis of β-catenin chromatin immunoprecipitation sequencing data identified angiogenesis-related gene regulation. Immunohistochemistry showed that higher microvessel density correlated with elevated nuclear β-catenin expression and unfavorable outcome. FH535 repressed the secretion of the proangiogenic cytokines vascular endothelial growth factor (VEGF), interleukin (IL)-6, IL-8, and tumor necrosis factor-α, and also inhibited angiogenesis *in vitro* and *in vivo*. Protein and mRNA microarrays revealed that FH535 downregulated the proangiogenic genes *ANGPT2, VEGFR3, IFN-γ, PLAUR, THPO, TIMP1,* and *VEGF*. FH535 not only represses pancreatic CSC stemness *in vitro*, but also remodels the tumor microenvironment by repressing angiogenesis, warranting further clinical investigation.

## INTRODUCTION

Pancreatic cancer is the fourth leading cause of adult cancer death, with a 5-year survival rate of only ~5%. The high mortality rate is due to its aggressive biological properties, late symptom onset, and failure of systemic therapies [1, 2]. Of all the pancreatic cancer treatments, radical surgery remains the only modality that can completely eradicate it [3]. However, the tumor is operable in only 5 ~ 25% of patients presenting with pancreatic cancer. Even after curative resection, the actual 5-year survival is only 10 ~ 20% [4]. Therefore, pharmacotherapeutics, although unsatisfactory, remain the main strategy for treating pancreatic cancer.

The WNT/β-catenin pathway is a genetically conserved signaling pathway associated with cell proliferation, migration, apoptosis, differentiation, and normal stem cell self-renewal [5]. In the absence of WNT ligands, β-catenin binds E-cadherin and forms complexes in the cell membrane. Free cytosolic β-catenin is recruited to a degradation complex constituted by anaphase-promoting complex (APC) protein, axin, and glycogen synthase kinase 3β (GSK3β). GSK3β phosphorylates β-catenin at certain key residues, leading to its ubiquitination and subsequent degradation. Upon stimulation, WNT ligands bind to the Frizzled/lipoprotein receptor–related protein 5/6 (FZD/LRP5/6) receptors and trigger inactivation of the degradation complex. Non-phosphorylated β-catenin accumulates in the cytoplasm, leading to nuclear translocation, where β-catenin interacts with T-cell factor transcription factors (TCF) and subsequently regulates the transcription of the downstream target genes [6, 7].

Aberrant WNT/β-catenin pathway activation plays a key role in regulating pathological carcinogenesis processes by facilitating tumor growth, migration, invasion, and contributing to cancer stem cell (CSC) maintenance [8]. Therefore, abnormal WNT/β-catenin pathway activation is closely related to the development of many cancers, including pancreatic cancer [9, 10]. Most reports to date support the association of the WNT/β-catenin pathway with several important factors determining the outcome of pancreatic cancer, where it causes extracellular matrix degradation and uncontrolled cell proliferation and differentiation [11], rendering the WNT/β-catenin pathway a promising target in pancreatic cancer treatment. As the nuclear distribution of β-catenin is associated with abnormal WNT/β-catenin pathway activation, we investigated the value of using nuclear β-catenin as a prognostic evaluation marker of pancreatic cancer.

Moreover, in previous reports, we demonstrated that FH535, a classic inhibitor of the β-catenin pathway, represses pancreatic cancer cell growth and metastasis *in vitro* [6, 7]. However, whether it could also have an anti–pancreatic cancer effect *in vivo* has not been explored. Therefore, in the present study, we investigated the anti-cancer effect of FH535 *in vivo* by using pancreatic cancer xenografts.

## RESULTS

### The β-catenin pathway is a promising target for treating pancreatic cancer

**Figure 1:**
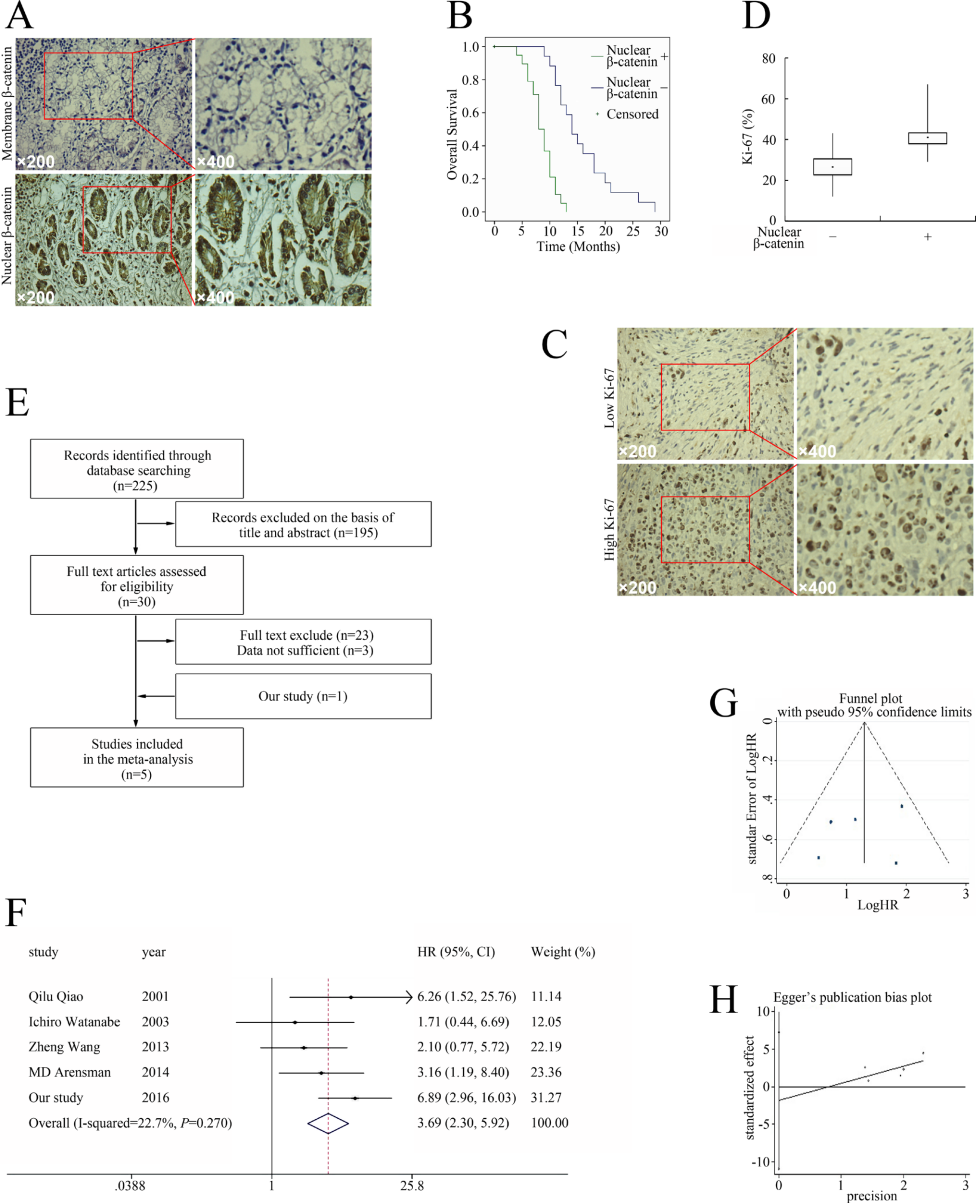
Relationship between aberrant β-catenin pathway activation and pancreatic cancer prognosis (**A**) Distribution of β-catenin in pancreatic cancer. (**B**) Nuclear β-catenin expression correlated with tumor characteristics and clinical outcomes of pancreatic cancer. Kaplan–Meier plot of OS stratified by nuclear β-catenin. (**C**) Ki-67 expression in pancreatic cancer. (**D**) The association of nuclear β-catenin and Ki-67 infiltration in pancreatic cancer cells. (**E**) Flow chart of studies included in the meta-analysis. (**F**) Meta-analysis of effects of β-catenin on OS in pancreatic cancer. (**G**) Funnel plot for the outcome of OS. (**H**) Egger's publication bias plot.

To determine the relationship between aberrant β-catenin pathway activation and the outcomes of pancreatic cancer, we measured the location of β-catenin in 58 tissue samples from patients with pancreatic cancer. Nuclear β-catenin was detected in 40/58 specimens (68.96%) (Figure 1A and Table 1); accordingly, the patients were divided into β-catenin–positive and -negative groups. Patients with nuclear β-catenin–positive tumors had shorter overall survival (OS) than the nuclear β-catenin–negative group (8.58 months vs. 15.82 months; *P* < 0.01, Figure 1B).

**Table 1:** The relationship between nuclear β-catenin expression and tumor characteristics and clinical outcomes of pancreatic cancers

Variable	*N* (%)	Nuclear β-catenin expression		χ^2^	*p*
		Positive (%)	Negative (%)		
Age (years)					
< 65	25 (43.10)	18 (31.03)	7 (12.07)	0.19	0.67
≥ 65	33 (56.90)	22 (37.93)	11 (18.97)		
Gender					
Male	37 (63.79)	26 (44.83)	11 (18.97)	0.08	0.78
Female	21 (36.21)	14 (24.14)	7 (12.07)		
OS (months)					
< 10	25 (43.10)	24 (41.38)	1 (1.72)	15.54	0.00
≥ 10	30 (51.72)	14 (24.14)	16 (27.59)		
Ki-67					
High	37 (63.79)	35 (60.34)	2 (3.45)	31.36	0.00
Low	21 (36.21)	5 (8.62)	16 (27.59)		

The mean Ki-67 percentage, a major prognostic factor of pancreatic tumors in general [12–14], was 37.48% (range 12–67%, Figure 1C). Based on the mean Ki-67, patients were divided into high Ki-67 (≥ 37.48%) and low Ki-67 groups (< 37.48%). The Ki-67 percentage in the nuclear β-catenin–positive group was 42.38% ± 7.85 as compared with the 26.61% ± 7.71 in the β-catenin–negative group (*P <* 0.01, Figure 1D). In the nuclear β-catenin–positive group, 70/80 patients (60.34%) had high Ki-67, while only 4/36 patients (3.45%) in the nuclear β-catenin–negative group had high Ki-67 (Table 1). Therefore, higher nuclear β-catenin levels predicted unfavorable outcome and correlated with elevated Ki-67, suggesting that the β-catenin pathway could be a potential target for treating pancreatic cancer.

We then performed a meta-analysis to confirm the relationship between aberrant β-catenin pathway activation and the outcome of pancreatic cancer on a larger scale. We screened 225 potentially eligible studies in the preliminary search (Figure 1E). Eventually, five studies (including the present study) [10, 15–17] investigating the correlation between β-catenin and OS in pancreatic cancer were included in the meta-analysis (Figure 1E). Table 2 outlines the major clinical characteristics of the enrolled studies. Although the studies all used immunohistochemical analysis to examine β-catenin expression, they used different criteria for β-catenin aberrant expression. Three studies directly reported HRs with 95% confidence intervals (CIs) [10, 16, 17], one study extrapolated them from Kaplan–Meier curves [15], and we calculated them from original data.

**Table 2:** Main characteristics of all studies included in the meta-analysis

Author	Year	Study location	Patient (male/female)	Age	Antibody source	Dilution	Method	Definition of abnormal β-catenin expression	Hazard ratio source	HR (95% CI)
Qiao et al.	2001	Germany	43 (23/20)	61.1 (42–83)	Transduction Laboratories	1:100	IHC	Cytoplasma	Calculated	6.26 (1.52–25.76)
Watanabe et al.	2003	Japan	23 (14/9)	58.1 (25–85)	Transduction Laboratories	1:100	IHC	Membranous reduced	Primary article	1.71 (0.44–6.69)
Wang et al.	2013	China	36 (25/11)	58.5 (34–76)	Santa Cruz	1:200	IHC	Positive cell	Primary article	2.10 (0.77–5.72)
Arensman et al.	2014	USA	41 (NR)	NR	NR	NR	IHC	Cytoplasma or nuclea	Primary article	3.16 (1.19–8.40)
Wu et al.	2015	China	58 (37/21)	65 (21–80)	Santa Cruz	1:100	IHC	Nuclear expression	Calculated	6.89 (2.96–16.03)

IHC, immunohistochemistry; NR, not reported; HR, hazard ratio.

The studies all reported the outcome of OS and had available data for calculating the HRs. As there was no significant heterogeneity among them (*P* = 0.27, *I*^2^ = 22.7%), a fixed-effects model was used to pool the data, and the results showed that aberrant β-catenin expression was associated with significantly increased mortality risk (HR 3.69, 95% CI 2.30–5.92, Figure 1F). The funnel plot did not reflect obvious asymmetry (Figure 1G), and the Egger test found no indication of publication bias (*t* = −1.847, *P* = 0.565 > 0.05, Figure 1H).

### FH535 had an anti-tumor effect against pancreatic cancer xenografts *in vivo*

**Figure 2:**
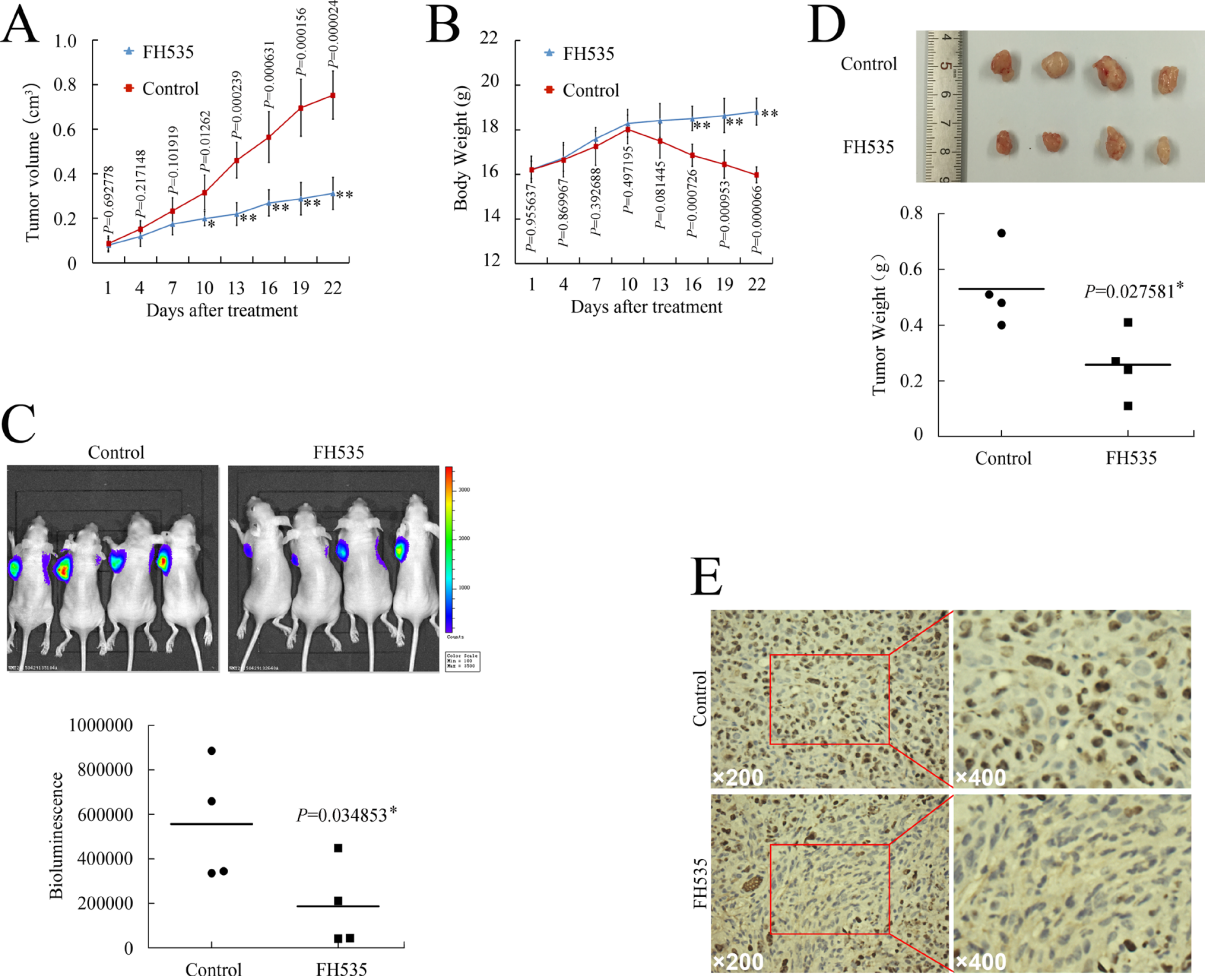
FH535 repressed pancreatic cancer xenograft growth *in vivo* (**A** and **B**) Tumor growth (A) and body weight (B) evaluation of FH535 vs. control group. (**C**) Representative *in vivo* bioluminescent images showing the effect of FH535 on pancreatic cancer. (**D**) Photographs of cancer xenograft and tumor weight evaluation in FH535 vs. control group. (**E**) Ki-67 expression in xenograft tumor sections from FH535- or DMSO-treated mice. *P < 0.05, **P < 0.01, significant differences vs. control group.

As aberrant β-catenin pathway activation predicted unfavorable outcomes, and strategies targeting the β-catenin pathway could be beneficial for treating pancreatic cancer, we evaluated the effect of FH535 on pancreatic cancer xenografts *in vivo*. On day 13 after the initiation of FH535 administration, the tumor volume of the FH535-treated group reached statistical significance as compared to the control group (Figure 2A). The control group mice had significant body weight loss, which could have been due to cancer-induced cachexia, while the FH535-treated mice showed better performance status (Figure 2B). At the termination of this study, FH535 had significantly suppressed tumor formation when detected with optical *in vivo* imaging technology (*P* < 0.05, Figure 2C). Thereafter, tumor samples were dissected from the mice (Figure 2D), and paraffin-embedded sections were prepared for immunohistochemical staining. FH535 repressed Ki-67 expression (Figure 2E). Therefore, FH535 could be a promising candidate for treating pancreatic cancer.

### FH535 impaired pancreatic cancer cell stemness

**Figure 3:**
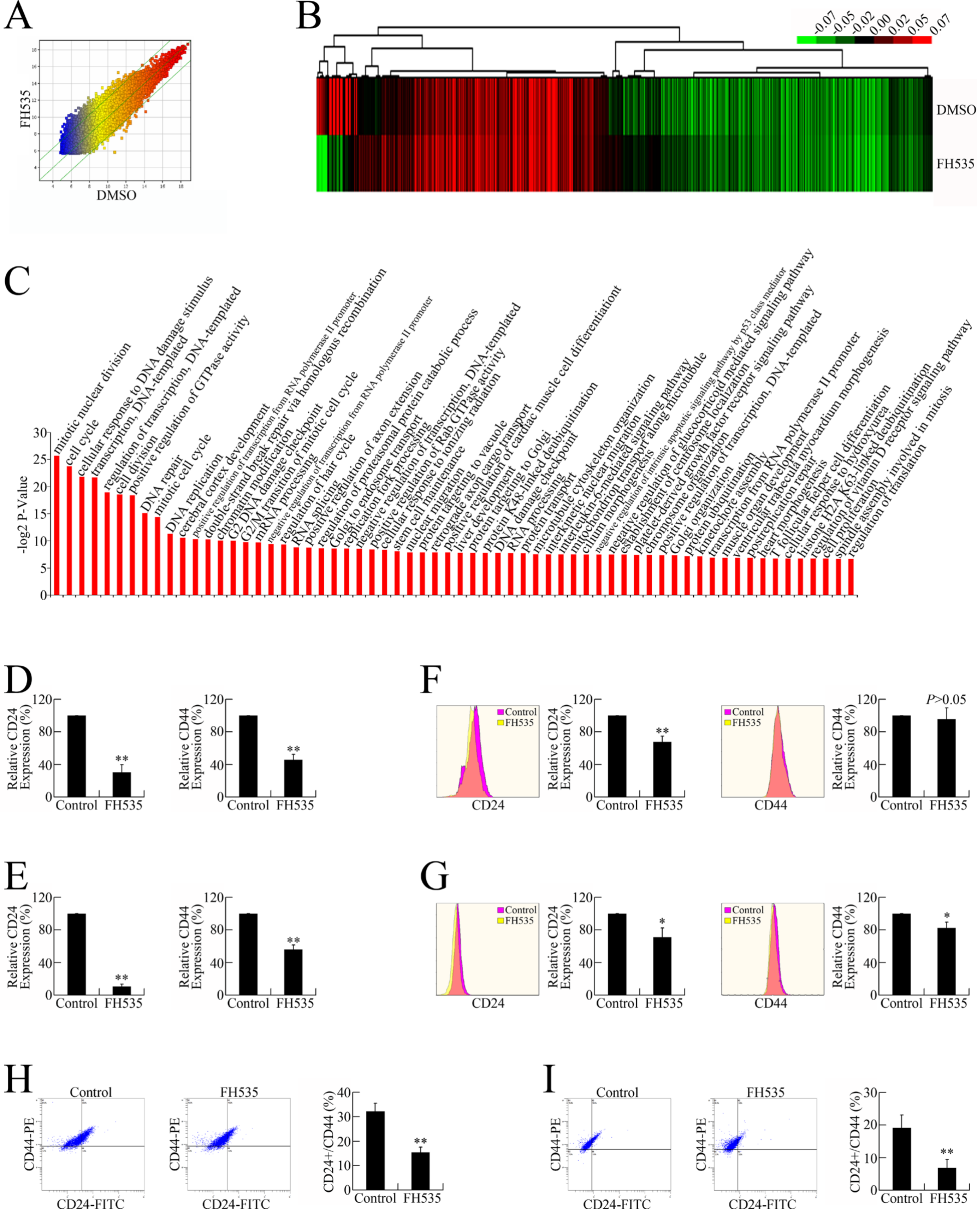
FH535 repressed pancreatic cancer cell stemness (**A**) Microarray analysis of the global expression profiles of control and FH535-treated groups. The scatter plot was used for assessing the gene expression variation (or reproducibility) between arrays. The X-axis (Control) and Y-axis (FH535) values in the scatter plot are the normalized signal values of the samples (log2 scaled). (**B**) Heat map of the differential gene expression patterns (fold change ≥ 1.5). (**C**) GO analysis of differentially expressed genes. (**D**) FH535 repressed *CD24* and *CD44* mRNA expression in PANC-1 cells. (**E**) FH535 repressed *CD24* and *CD44* mRNA expression in CFPAC-1 cells. (**F**) FH535 repressed protein expression of CD24 but not CD44 in PANC-1 cells. (**G**) FH535 repressed CD24 and CD44 protein expression in CFPAC-1 cells. (**H**) FH535 downregulated the PANC-1 cell CD24+/CD44+ subgroup. (**I**) FH535 decreased the CFPAC-1 cell CD24+/CD44+ population.

Microarray analyses were conducted to profile global gene expression patterns (Figure 3A and 3B); Gene Ontology (GO) analyses were then performed for the differentially expressed genes. The 685 annotated genes encode a wide range of functions, including cell cycle, cell division, DNA damage and repair, DNA replication, cell proliferation, and stem cell maintenance (Figure 3C and Supplementary Table S1).

As GO analyses indicated biological processes related to stemness maintenance, we speculated that the anti-cancer effect of FH535 might involve repressing pancreatic CSC stemness. To verify this hypothesis, the expression of CD24 and CD44, two pancreatic CSC markers, were evaluated in FH535-treated pancreatic cancer cells. FH535 repressed the mRNA and protein expression of both CD24 and CD44 (Figure 3D–3G), leading to downregulated CD24+/CD44+ populations (Figure 3H and 3I), the presumed pancreatic CSCs [18, 19].

### Chromatin immunoprecipitation sequencing (ChIP-Seq) data analysis and identification of β-catenin–targeted genes

**Figure 4:**
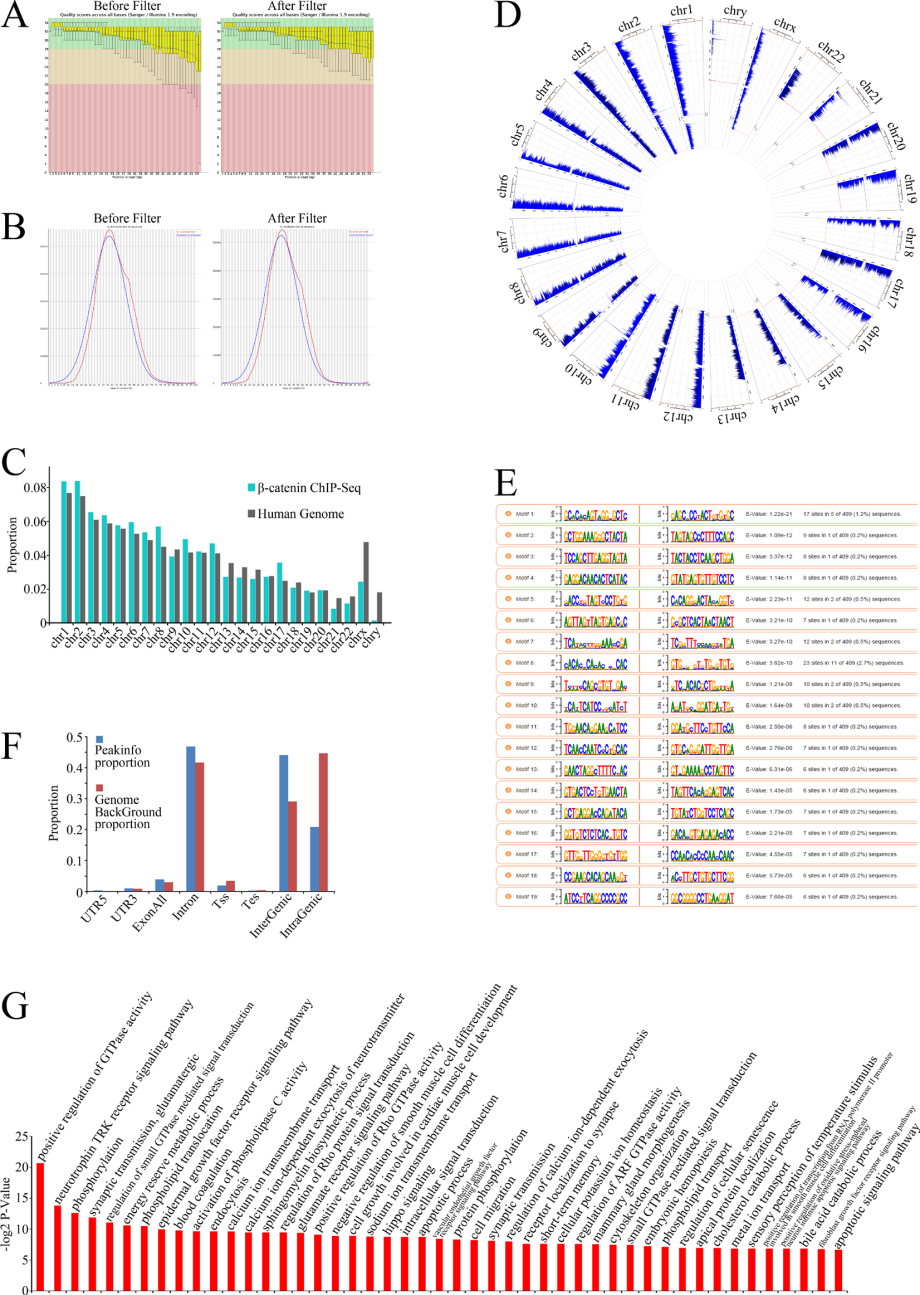
β-Catenin ChIP-Seq data analysis (**A**) Box plots of base call quality scores across all bases before (left) and after (right) filtering were obtained using an Illumina GA II sequencer. Green and red areas, quality scores > 28 and < 20, respectively. Yellow boxes, upper and lower quartiles; whiskers, 10% and 90% quartiles. Red horizontal lines, median value. Blue curves, mean quality scores. Quality scores were given based on Phred-scaled quality values calculated using *q* = −10log10(P), with P being the estimated error probability for that base call. (**B**) Overview of mean GC distribution over all sequences for samples before (left) and after (right) filtering. Red curves, GC content per read; blue curves, theoretical distribution. Horizontal axis corresponds to mean GC content (%); vertical axis corresponds to GC content. (**C**) Proportion of ChIP-Seq reads uniquely mapped to each chromosome. (**D**) Chromosomal distribution of ChIP-Seq reads across all chromosomes. (**E**) Proportion of ChIP-Seq reads uniquely mapped to eight human genomic regions: 5′UTR (untranslated region), CDS (coding sequence), 3′UTR, exon all, introns, Tss (transcription start site), Tes (transcription end site), intergenic or intragenic regions. (**F**) Motif analysis of ChIP-Seq data. The nucleotide frequencies of the genomic sequences aligned at the top-scoring 15 motifs are shown in a sequence logo representation. (**G**) GO analysis of identified target genes.

To identify the genes directly regulated by β-catenin, we analyzed the β-catenin ChIP-Seq data. Tags that were mapped to multiple locations and tags with low-quality scores were filtered out (Figure 4A and 4B). Figure 4C and 4D present the reads distribution on chromosomes. Peak calling programs were performed to define protein:DNA binding sites by identifying regions where sequence reads were enriched in the genome after mapping (Supplementary Table S2 and Supplementary Table S3). Motif analysis was performed to identify the most frequent binding sites (Figure 4E and Supplementary Table S4).

To annotate the biological processes–related β-catenin target genes, binding sites derived from peak calling (*P* < 0.001) ounderwent GO analyses. The annotated genes exhibited a wide range of functions related to oncogenic activity, including phosphorylation, the epidermal growth factor receptor (EGFR) signaling pathway, the apoptotic process, cell migration, the vascular endothelial growth factor receptor (VEGFR) signaling pathway, cell cycle arrest, and regulation of extracellular matrix organization (Figure 4F and Supplementary Table S5), suggesting that the genes not only regulate cellular biological processes themselves, but also participate in cancer microenviroment remodeling, such as angiogenesis and extracellular matrix organization.

### FH535 repressed pancreatic cancer angiogenesis *in vitro* and *in vivo*

**Figure 5:**
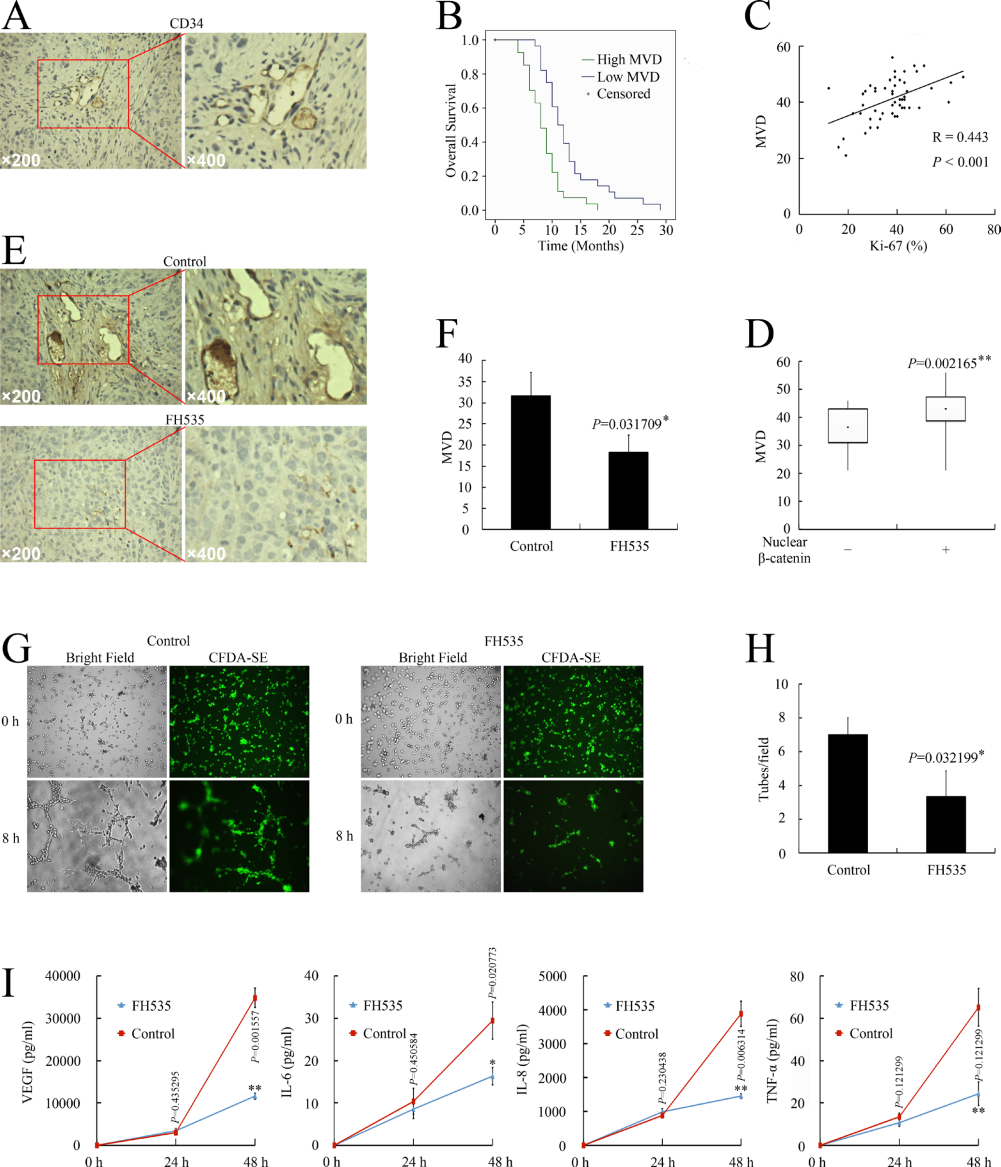
Nuclear β-catenin expression correlated with angiogenesis in pancreatic cancer (**A**) CD34 expression in pancreatic cancer cells. (**B**) Kaplan–Meier plot of OS stratified by MVD. (**C**) Association of MVD with Ki-67 infiltration. (**D**) Association of nuclear β-catenin with MVD. (**E**) Immunohistochemical examination of pancreatic orthotopic xenografts by using CD34 antibody. (**F**) FH535 treatment decreased MVD level in pancreatic orthotopic xenografts. (**G**) Images of HUVEC tube formation assay for angiogenesis *in vitro.* (**H**) HUVEC tube formation assay evaluation of FH535 inhibition of angiogenesis *in vitro*. *P < 0.05, significant differences vs. control group. (**I**) Milliplex assay of secreted proangiogenic factors in culture medium after treatment with 20 μM FH535. *P < 0.05, **P < 0.01, significant differences vs. the respective control groups.

As β-catenin target genes participate in the regulation of vascularizationon, we investigated whether aberrant WNT/β-catenin pathway activation is correlated with angiogenesis in pancreatic cancer, investigating the relationship between nuclear β-catenin expression and angiogenesis in pancreatic cancer specimens. CD34 was detected in all 58 specimens. All endothelial cells in the tissue were positively stained by the CD34 antibodies (Figure 5A). The microvessel density (MVD) was 41.12 (range 21–56). Based on the mean MVD, patients were divided into high MVD (≥ 41.12) and low MVD groups (*<* 41.12). Patients with higher MVD had shorter OS (8.63 months vs. 12.93 months; *P <* 0.01), suggesting that high MVD is predictive of poor prognosis and shorter survival in human pancreatic cancer (Figure 5B).

Spearman correlation analysis confirmed the positive correlation between MVD and Ki-67 level (*R* = 0.433, *P <* 0.001, Figure 5C). In the high MVD group, 26/29 patients (44.83%) had high Ki-67 (≥ 37.48%) while only 11/29 patients (18.97%) in the low MVD group had high Ki-67 (*P <* 0.01, Table 3).

**Table 3:** The relationship between MVD and tumor characteristics and clinical outcomes of pancreatic cancers

Variable	*N* (%)	MVD		χ^2^	*p*
		High	Low		
Age (years)					
< 65	25 (43.10)	11 (18.97)	14 (24.14)	0.63	0.42
≥ 65	33 (56.90)	18 (31.03)	15 (25.86)		
Gender					
Male	37 (63.79)	21 (36.21)	16 (27.59)	1.87	0.17
Female	21 (36.21)	8 (13.79)	13 (22.41)		
OS (months)					
< 10	25 (43.10)	17 (29.31)	8 (13.79)	7.90	0.00
≥ 10	30 (51.72)	9 (15.52)	21 (36.21)		
Nuclear β-catenin					
Positive	40 (68.97)	24 (41.38)	16 (27.59)	5.16	0.02
Negative	18 (31.03)	5 (8.62)	13 (22.41)		
Ki-67					
High	37 (63.79)	26 (44.83)	11 (18.97)	16.80	0.00
Low	21 (36.21)	3 (5.17)	18 (31.03)		

Next, we analyzed the correlation between nuclear β-catenin expression and MVD. The MVD in the nuclear β-catenin–positive group was 43.28 ± 5.75 compared with the 36.33 ± 7.74 in the nuclear β-catenin–negative group (*P <* 0.05, Figure 5D). In the nuclear β-catenin–positive group, 24/40 patients (41.38%) had high MVD while only 5/18 patients (8.62%) in the nuclear β-catenin–negative group had high MVD (*P <* 0.05, Table 3).

As higher β-catenin pathway activity correlated with elevated angiogenesis in the tissue samples, we further investigated whether FH535 could repress angiogenesis in a pancreatic cancer model. FH535 repressed MVD in pancreatic cancer xenografts (*P* < 0.05, Figure 5E and 5F). The human umbilical vein endothelial cell (HUVEC) tube formation potency of supernatant obtained from FH535-treated PANC-1 cells was also significantly reduced (*P* < 0.05, Figure 5G and 5H). Therefore, FH535 repressed pancreatic cancer angiogenesis both *in vitro* and *in vivo*. Consistent with the repressed angiogenetic potency, FH535 also significantly decreased the levels of pancreatic cancer cell–secreted pro-angiogenic VEGF, interleukin (IL)-6, IL-8, and tumor necrosis factor (TNF)-α (Figure 5I).

### FH535 repressed the expression of angiogenesis-related genes in pancreatic cancer cells

**Figure 6:**
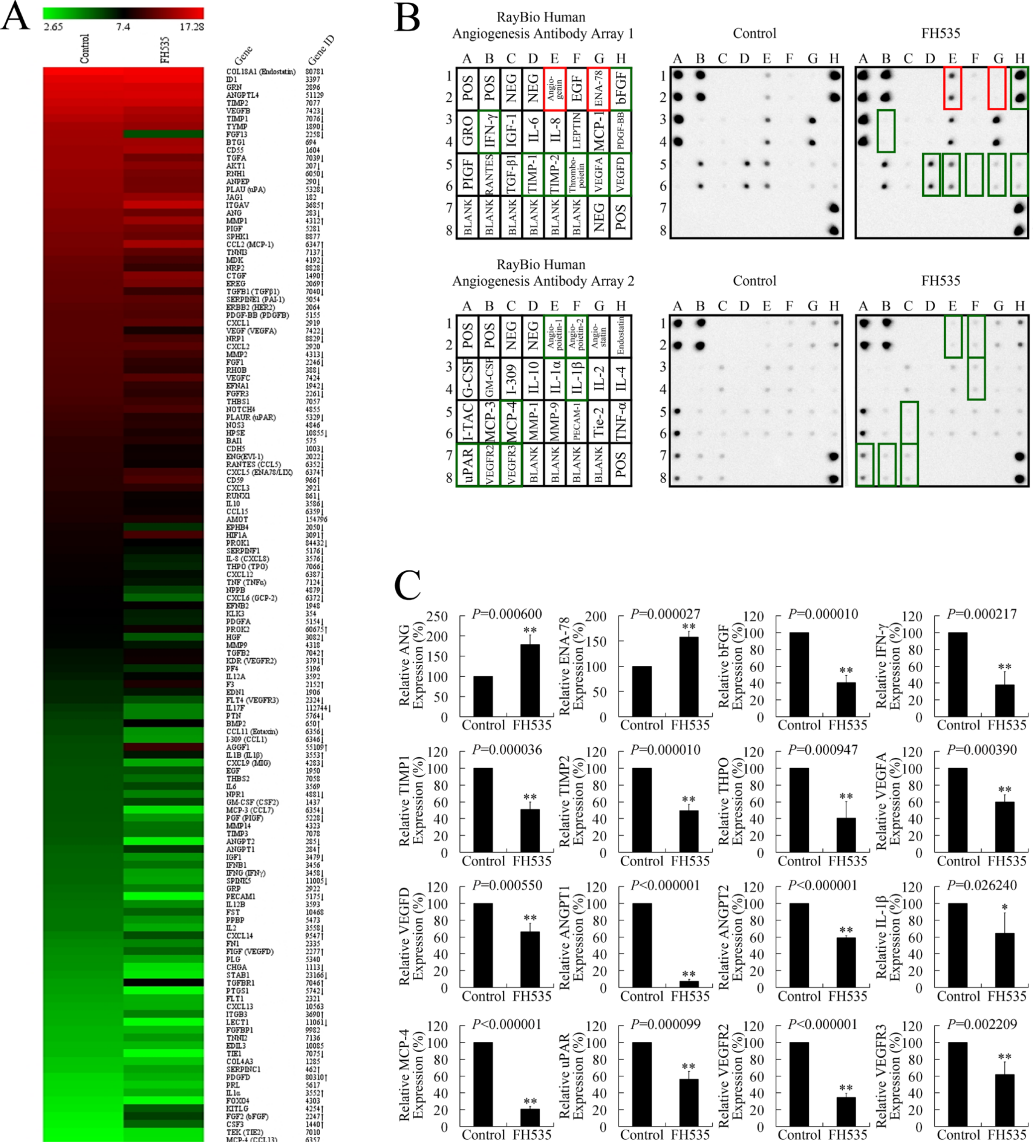
FH535 repressed angiogenesis-related genes in pancreatic cancer cells (**A**) Microarray analysis of angiogenesis-related gene expression regulation upon treatment with 20 μM FH535. Up and down arrows, gene expression significantly upregulated or downregulated, respectively, by 1.5-fold. (**B**) Antibody arrays of protein expression of angiogenesis-related genes after FH535 treatment. (**C**) Analysis of antibody array grey levels. *P < 0.05, **P < 0.01, significant differences vs. the respective control groups.

To investigate the mechanisms involved in FH535 repression of angiogenesis, we analyzed microarray data to illustrate the expression of angiogenesis-related genes following FH535 treatment in pancreatic cancer cells (Figure 6A). The provascularization *VEGF* (VEGFA), *VEGFB*, *FL*T4 (VEGFR3), *ANG* (angiogenin), *ANGP*T2, *AKT1*, *PLAU* (urokinase, uPA), *PLAUR* (uPA receptor, uPAR), *IL8* (CXCL8), *IL10*, *IL17F*, *TGFA*, *TGFB1* (transforming growth factor [TGF]β1), *FGF1*, *FGF13*, *FGFR3*, *PGF* (PIGF), *HGF*, *IGF1*, *PDGFA*, *THPO* (thyroid peroxidase, TPO), *TNF* (TNF-α), *IFN-γ* (interferon [IFN]-γ), *TIMP1*, and *MMP2* were downregulated at mRNA level. We also evaluated the protein expression of the angiogenesis-related genes after FH535 treatment. FH535 downregulated the expression of proangiogenic bFGF (fibroblast growth factor [FGF]2), IFN-γ, tissue inhibitor of metalloproteinase (TIMP)1, TIMP2, THPO, VEGFA, VEGFD, angiopoietin (ANGPT)1, ANGPT2, IL-1β, monocyte chemoattractant protein (MCP)-4, uPAR, VEGFR2, and VEGFR3 protein (Figure 6B and 6C). By cross-comparing the two microarray datasets, we determined that FH535 repressed *ANGPT2*, *FLT4*, *IFN-γ*, *PLAUR*, *THPO*, *TIMP1*, and *VEGF* expression at both mRNA and protein level. Therefore, it is possible that the antiangiogenic effect of FH535 is executed through a mechanism involving multiple genes.

## DISCUSSION

WNT/β-catenin signaling pathway activity is essential for embryonic development, and its dysregulation appears to be one of the major drivers of pancreatic carcinogenesis [20]. Although β-catenin accumulation is not a universal characteristic of the disease, both nuclear and cytoplasmic accumulation of β-catenin are observed in pancreatic cancer [21–23]. Accumulating functional evidence also implicates a supporting role for β-catenin in pancreatic cancer maintenance and progression [24]. There is also emerging evidence that β-catenin accumulation and signaling could be increased through paracrine signaling, which occurs in the pancreatic cancer microenvironment. Our present results suggest a pathologic role for nuclear β-catenin in promoting pancreatic cancer growth. Analysis of the pancreatic cancer samples revealed that high nuclear β-catenin expression correlated significantly with shorter OS.

Although many studies have explored the correlations between β-catenin expression and the prognosis of pancreatic cancer, the conclusions have been inconsistent. Previous meta-analyses have proven that nuclear β-catenin overexpression predicts progressive disease and unfavorable survival in colorectal cancer [25], lung cancer [26, 27], hepatocellular cancer [28], breast cancer [29], esophageal cancer [30], and gastric cancer [31]. As the prognostic value of β-catenin in pancreatic cancer remains controversial, a meta-analysis was needed to explore the issue clearly. We pooled the survival data of 201 patients with pancreatic cancer from five studies (including the present study) and found that aberrant β-catenin expression was associated with significantly increased mortality risk (HR 3.69, 95% CI 2.30–5.92). Our meta-analysis revealed that aberrant β-catenin expression could be a predictive factor of poor prognosis of pancreatic cancer and that the WNT/β-catenin pathway could be a promising treatment target of pancreatic cancer.

Currently, the pancreatic CSC markers include CD24, CD44, epithelial cell adhesion molecule (EPCAM) [32], c-MET [33], CD133 [34], aldehyde dehydrogenase (ALDH) [34], and Hoechst dye exclusion (side population) [35]. Combination, but not single, markers have been used to identify pancreatic CSCs. CSCs. have been proven to preserve the abilities of extensive proliferation, self-renewal, multi-lineage differentiation, and high tumorigenic potential [36]. A recent study using breast cancer biopsy tissues showed that chemotherapy led to an increased percentage of CD44+/CD24+ cells, consistent with increased clone formation ability [37]. CD24+/CD44+ pancreatic cancer cells have a significantly higher possibility for forming colonies *in vitro* and are more resistant to irradiation [18]. Survival analysis has shown that CD24+/CD44+ expression appears to be correlated with poor prognosis [19]. Therefore, CD24 and CD44 are now widely used for identifying pancreatic CSCs [19, 34, 38–40]. It has been well established that the WNT/β-catenin signaling pathway is crucial for normal stem cell self-renewal and tissue differentiation [5], while aberrant WNT/β-catenin signaling pathway activation contributes to the maintenance of CSCs [8, 41] n. Previously, we found that FH535 induced cell cycle arrest and repressed pancreatic cancer cell growth *in vitro*. Presently, we discovered that FH535 decreased the population of CD24+/CD44+ pancreatic cancer cells, the presumed pancreatic CSCs. Therefore, we speculated that the anti-cancer effect of FH535 could also involve repressing CSC stemness.

*In vivo*, cancer cells are surrounded by a complex milieu. This cancer cell niche is called the tumor microenvironment, and contributes to tumor development and metastasis [42]. Angiogenesis has been proven to be a crucial influencing factor in the tumor microenvironment; it is the foundation of solid tumor growth and metastasis. It is well established that all successful tumors must undergo neovascularization in order to acquire nutrients for continued growth and metastatic spread. Without angiogenesis, a solid tumor rarely grows larger than 2 ~ −3 mm [43]. Research on angiogenesis in general is a major focus in biomedicine and has led to the clinical approval of several antiangiogenic agents, including thalidomide, bevacizumab, sorafenib, sunitinib, pazopanib, temsirolimus, and everolimus. Indeed, antiangiogenic agents have significantly changed treatment strategies for solid tumors, including colorectal cancer, renal cell carcinoma, and breast cancer [44]. However, whether antiangiogenic therapeutics is a promising strategy for treating pancreatic cancer still requires verification.

In fact, increasing evidence indicates that WNT/β-catenin signaling plays a pivotal role in tumor angiogenesis by inducing endothelial cell proliferation and survival [8, 45]. Studies have revealed that WNT overexpression is associated with the expression of genes that participate in angiogenesis [46–52]. Furthermore, the WNT/β-catenin pathway WNTfrequently cooperates with the other angiogenesis-related pathways such as the Notch, mitogen-activated protein kinase (MAPK), and nuclear factor (NF)-κB pathways [53]. For example, the crosstalk between the WNT/β-catenin and Notch pathways mediates vascular angiogenesis and endothelial specification [54]. Secreted Frizzled-related proteina WNT inhibitor inhibits angiogenesis,, by binding WNT protein and antagonizing WNT/β-catenin signaling, inversely suggesting that WNT protein may be implicated in vascular proliferation [55]. Presently, we investigated whether a β-catenin pathway–targeting therapeutic could exert an antiangiogenic effect in pancreatic cancer.

MVD is the best recognized indicator for evaluating angiogenesis in solid tumors [56]. Immunostaining of a vascular endothelial cell marker such as CD34 is used to evaluate MVD [57–60]. MVD is an adverse prognostic factor in pancreatic cancer [61] and several other cancers [57, 58, 62, 63]. Via CD34 staining, we not only proved the association between MVD and the prognosis of pancreatic cancer, but also determined the relationship between β-catenin and angiogenesis, suggesting that the *in vivo* anti-cancer effect of a β-catenin pathway inhibitor could involve an antiangiogenic function targeting the tumor microenvironment. The HUVEC tube formation assay further showed that FH535 repressed the proangiogenic ability of pancreatic cancer cells. Moreover, FH535 decreased MVD in pancreatic cancer xenografts. Therefore, a β-catenin pathway–targeting therapeutic such as FH535 represses the angiogenetic potency of pancreatic cancer both *in vitro* and *in vivo*.

Abundant angiogenesis regulators and oncogene activation are essential for an angiogenesis phenotype that supports tumorigenicity [64]. ProteinM and mRNA microarrays revealed that FH535 significantly suppressed the expression of seven proangiogenic genes: *ANGPT2*, *FLT4* (VEGFR3), *IFN-γ*, *PLAUR* (uPAR), *THPO* (TPO), *TIMP1*, and *VEGF* (VEGFA), at both mRNA and protein level. Moreover, the Milliplex assay showed that FH535 significantly decreased VEGF, IL-6, IL-8, and TNF-α levels in culture medium, indicating that the WNT/β-catenin pathway–targeting strategy represses angiogenesis in pancreatic cancer through a mechanism involving multiple genes. It is worth noting that FH535 repressed VEGF at mRNA expression, and protein expression and secretion levels. As VEGF plays a central role in angiogenesis, its repression could be critical to the manner in which FH535 exerts its antiangiogenic effect.

Taken together with our previous studies, we proved that FH535 exerts an anti-cancer effect against pancreatic cancer, targeting pancreatic cancer cells by repressing cell growth, metastasis, and stemness and targeting the tumor microenvironment by inhibiting angiogenesis. FH535 exerted its anti-cancer effect against pancreatic cancer xenografts *in vivo* and presents the possibility of clinical application. Although targeting the WNT signaling pathway in the clinic using small-molecule therapeutics or biologics is still in its infancy, several therapeutics targeting the WNT/β-catenin pathway have already progressed from bench to bedside [65]. For example, OMP-18R5 is a fully humanized monoclonal antibody that binds to FZD [66]; OMP-54F28 is an Fc fusion protein with FZD8 that binds all WNT ligands [67]; LGK974 inhibits Porcupine, which enables WNT secretion [68, 69]; PRI-724 is a specific CREB-binding protein (CBP) and β-catenin antagonist [65]. Clinical trials have been initiated to evaluate the effectiveness of these therapeutics against pancreatic cancer [65, 67]. Although FH535 appears tolerable in mouse models, its on-target toxicity (i.e., WNT inhibition and effects on intestinal stem cells, bone turnover, and hematopoiesis [67]) and off-target toxicity (i.e., inhibition of other members of this class of *O*-acyltransferases or similar enzymes [67]) still requires careful evaluation in further investigations.

## MATERIALS AND METHODS

### Ethics statement

This investigation has been conducted in accordance with the ethical standards and according to the Declaration of Helsinki and according to national and international guidelines and has been approved by the First Affiliated Hospital of Soochow University Committee on Medical Ethics Institutional Review Board.

### Patients and tissue samples

The study material was obtained from 58 patients with pancreatic cancer whose tissue samples were available (mean age 65 years, range 21 ~ 80 years) and who were treated during January 2007 to October 2013 at the First Affiliated Hospital of Soochow University. All human tissue samples were obtained and handled in accordance with an approved Institutional Review Board application (the Committee on Medical Ethics, the First Affiliated Hospital of Soochow University). Tumor characteristics were obtained from the pathology database (Table 1). Patients received systemic therapy according to National Comprehensive Cancer Network pancreatic cancer clinical practice guidelines and were followed regularly for 2 years. Prognostic analyses were performed regarding OS.

### Meta-analysis

We carried out a search of the PubMed database using the following terms and all possible combinations: “pancreatic cancer”, “pancreatic carcinoma”, “pancreatic neoplasm”, “beta-catenin”, “β-catenin” and “CTNNB1”. The search duration was from November 1985 to November 2015; no lower date limit was used. The citation lists associated with the studies were used to identify additional eligible studies. Reviews and bibliographies were also manually inspected to find related articles. The inclusion criteria, exclusion criteria, and statistical analysis are presented in the Supplementary Methods.

### Cell lines and culture conditions

The human pancreatic cancer cell lines PANC-1 and CFPAC-1, and HUVECs, were purchased from American Type Culture Collection and maintained in a humidified incubator with a 5% CO_2_–95% air atmosphere at 37°C. PANC-1 and CFPAC-1 cells were maintained in Dulbecco's modified Eagle's medium (DMEM) supplemented with 10% FBS (Gibco), 100 units/mL penicillin, and 100 μg/mL streptomycin. HUVECs were maintained in DMEM supplemented with 10% FBS, 2 mmol/L glutamine, 0.1 mmol/L hypoxanthine, 0.4 mmol/L aminopterin, 16 mmol/L thymidine, 100 units/mL penicillin, and 100 μg/mL streptomycin.

### Immunohistochemistry

All resection specimens were fixed in 10% buffered formalin and paraffin-embedded by routine processing. Sections were cut at 4-μm thickness, heated at 60°C for 30 min, then deparaffinized and hydrated through a series of xylene and alcohol baths. The slides were microwaved with antigen retrieval solution (citrate buffer, pH 6.0, containing 0.3% trisodium citrate and 0.04% citric acid) for 5 min. After replenishment of this solution, the slides were microwaved again for 5 min and then allowed to cool for 20 min. The sections were then rinsed in PBS and immersed in 3% H_2_O_2_ for 15 min to block the endogenous peroxidase. Thereafter, the sections were incubated with 10% bovine serum albumin at room temperature for 1 h to block nonspecific antibodies. Immunohistochemical staining was performed with mouse anti–β-catenin antibody (sc59737; Santa Cruz Biotechnology), mouse anti–Ki-67 antibody (GM724029; GeneTech, Shanghai, China), or rabbit anti-CD34 antibody (ab81289; Abcam, London, UK) at room temperature for 2 h. After incubation with the corresponding secondary antibodies for 20 min, the bound complex was visualized using a SuperPicture Polymer Detection Kit (No. 87-8963; Invitrogen).

### Evaluation of immunostaining

Two independent researchers evaluated the immunostaining results. All analyses were performed blind with respect to the clinical outcomes. Briefly, the five most representative high-power fields (×400 magnification) per slide were selected. To evaluate the validity of the analysis, the area measurement and counting were repeated 4 weeks later.

To grade the immunohistochemical findings for β-catenin, sections were scored based on the extent and intensity of staining; membranous and cytoplasmic staining were assessed separately [10]. The scoring system is presented in the Supplementary Methods. Ki-67 immunoreactivity was expressed as the mean percentage of tumor cells with the highest nuclear labeling after manual counting of 20 hotspot areas. Endothelial cells were marked with anti-CD34 antibody and subjected to MVD and angiogenesis vascularity evaluation [70] (Supplementary Methods).

### Nude mouse tumor xenograft model and treatment

Four-week-old female BALB/c athymic nude mice were purchased from Shanghai SLAC Laboratory Animal Co. Ltd (Shanghai, China) and received humane care according to the Soochow University Institutional Animal Care and Treatment Committee. PANC-1 cells stably expressing firefly luciferase were injected into the left flanks of the mice in a total volume of 100 μL (0.5 × 10^7^ cells), and the mice were randomly assigned to a dimethyl sulfoxide (DMSO) [intraperitoneally injected with 100 μL DMSO/DMEM (1:1)] or FH535 group [intraperitoneally injected with 25 mg/kg FH535 (Millipore) dissolved in 100 μL DMSO/DMEM (1:1)]. Treatment was conducted every 2 days for 20 days; tumor volume was measured with a caliper using the formula: volume = length × width^2^/2. At the end of the experiment, the mice were anaesthetized and given D-luciferin in PBS. Twenty minutes after the injection, bioluminescence was imaged with a charge-coupled device camera (IVIS; Lumina II, PerkinElmer). Then, the tumor tissue was stripped and formalin-fixed, paraffin-embedded, cut into 4-μm sections, and immunohistochemically stained.

### Microarray assay

Total RNA from each sample was amplified and transcribed into fluorescent cRNA according to Agilent Technologies' Quick Amp Labeling protocol (version 5.7). The labeled cRNAs were hybridized onto a Whole Human Genome Oligo Microarray (4×44K; Agilent Technologies). After washing the slides, the arrays were scanned using an Agilent Technologies Scanner G2505C. Agilent Technologies Feature Extraction software (version 11.0.1.1) was used to analyze the acquired array images. Quantile normalization and subsequent data processing were performed using the GeneSpring GX v11.5.1 software package (Agilent Technologies). Differentially expressed genes were identified through fold change filtering. The selection criterion was > 1.5-fold difference in expression (upregulated expression > 1.5-fold; downregulated expression < 0.67-fold). GO analysis (Supplementary Methods) was used to determine the roles played by the differentially expressed genes in these biological GO terms.

### Real-time PCR

Total RNA was extracted using TRIzol (Invitrogen) according to the manufacturer's protocol. After spectrophotometric quantification, 1 μg total RNA in a final volume of 20 μL was used for reverse transcription with a PrimeScript RT Reagent Kit (TaKaRa, Otsu, Shiga, Japan) according to the manufacturer's protocol. Aliquots of cDNA corresponding to equal amounts of RNA were used for mRNA quantification by real-time PCR using a LightCycler 96 Real-time Quantitative PCR Detection System (Roche). The reaction system (25 μL) contained the corresponding cDNA, forward and reverse primers, and SYBR Green PCR master mix (Roche). All data were analyzed using *B2M* gene expression as the internal standard. The specific primers were as follows: (1) *CD24*, forward, 5′-CAGGGCAATGATGAATGAGAAT-3′, reverse, 5′-CC TGGGCGACAAAGTGAGA-3′, product, 233 bp; (2) *CD44*, forward, 5′-GTGATGGCACCCGCTATGTC-3′, reverse, 5′-AACCTCCTGAAGTGCTGCTCC-3′, product, 129 bp; (3) *B2M*, forward, 5′-TCAAGAAGGTG GTGAAGCAG-3′, reverse, 5′-AAGGTGGAGGAGT GGGTGTC-3′, product, 112 bp.

### Evaluation of protein expression

The expression levels of CD24 and CD44 protein in pancreatic cancer cells was measured by flow cytometry. Following treatment, the cells were harvested, fixed with 4% paraformaldehyde, and permeabilized using 0.1% Triton X-100. After washing thrice with PBS, the cells were incubated with anti-CD24 (fluorescein isothiocyanate [FITC]-conjugated; Santa Cruz Biotechnology) and anti-CD44 (phycoerythrin [PE]-conjugated, Santa Cruz Biotechnology) antibodies for 30 min at 4°C. Subsequently, the cells were analyzed using a Beckman Coulter FC500 flow cytometer.

### Stemness evaluation of pancreatic cancer cells

The cells were harvested, washed twice with 2% FBS/PBS solution, and resuspended in 100 μL 2% FBS/PBS before incubating with FITC-conjugated anti-CD24 and PE-conjugated anti-CD44 antibodies for 30 minutes at 4°C. The cells were then washed twice with 2% FBS/PBS solution and resuspended in 500 μL 2% FBS/PBS prior to reading on a Beckman Coulter FC500 flow cytometer.

### ChIP-Seq data analysis

β-Catenin ChIP-Seq data (SRX017112) were obtained from the Sequence Read Archive database (http://www.ncbi.nlm.nih.gov/sra/SRX017112). The raw sequencing data were evaluated by FAST-QC, including quality distribution of nucleotides, position-specific sequencing quality, GC content, proportion of PCR duplication, and Wmer frequency. We used Burrows-Wheeler Aligner, a backward search–based read alignment package, with Burrows–Wheeler transform for genome-wide mapping. Peak calling was performed comparing ChIP material to input using MACS14 (1.4.0rc2); binding regions were ranked based on sequencing tag enrichment by comparing each ChIP library to the control. A threshold *P*-value < 0.0001 was applied; alignment was carried out using FastQC with hg19_GRCh37 as the build. Motifs were discovered using XXmotif (http://xxmotif.genzentrum.lmu.de), a *de novo* motif discovery method that directly optimizes the statistical significance of position weight matrices [71, 72].

### Tube formation assay for angiogenesis evaluation

For basement membrane reconstitution, Matrigel (BD Biosciences) was diluted 2-fold with cold DMEM (without FBS) and added to 96-well plates at 4°C. The plates were incubated for 2 h in a 37°C cell culture incubator to allow the Matrigel to solidify. Carboxyfluorescein diacetate succinimidyl ester (Beyotime, Shanghai, China)-labeled HUVECs were trypsinized, counted, resuspended in basal medium, and added on top of the reconstructed basement membrane (5 × 10^4^ cells/well). After 4~12 h, the formed networks were photographed and analyzed using Image-Pro Plus (Media Cybernetics) to calculate the area of the network. Endotubes were quantified by counting nine random fields/sample under a microscope (×40 magnification).

### Milliplex assay

A multiplex biometric immunoassay containing fluorescent-dyed microbeads (Cat. No. HCYTOMAG-60 K; Millipore) was used for measuring VEGF, IL-6, IL-8, and TNF-α levels in culture medium. Mean fluorescence intensity was calculated by Luminex Technology (Bio-Plex Workstation; Bio-Rad Laboratories). Data were analyzed using Milliplex^®^ Analyst 5.1 (Bio-Rad Laboratories).

### Angiogenesis antibody array

Proteins in the cell samples were biotinylated, and spin-filtered to remove excess biotin. The biotinylated samples were dialyzed with PBS and added to RayBio Human Angiogenesis Antibody Array membranes (RayBiotech, Norcross, Ga). Biotinylated proteins captured by the membrane-bound antibodies were detected by incubation with horseradish peroxidase–streptavidin and analyzed using a chemiluminescence imaging system (RayBiotech). Normalization was performed using the signal of internal controls on each protein array chip. Quality control was performed by removing proteins detected below the raw signal intensity of 50, which was twice the maximum intensity of the negative control probes. Significance testing was performed by *t*-test; fold-change was cut-off at 1.5.

### Statistical analysis

Statistical analysis was performed using SPSS 16.0 software (SPSS Inc.). Chi square tests were used for comparing Ki-67, MVD, and β-catenin. The correlations between MVD and Ki-67 were analyzed using Spearman's rank test. Kaplan–Meier curves were constructed to analyze survival data; statistical analysis was carried out using log-rank testing. OS was defined as the time from the beginning of surgery to death from any cause or the last date of follow-up. The relationships between nuclear β-catenin or MVD and tumor characteristics or clinical outcomes were examined and assessed using univariate analysis. For gene expression data, statistical analyses were carried out with unpaired Student's *t*-tests. All data are presented as the mean ± SD. Statistical significance was determined at *P* < 0.05; all tests were two-sided.

## SUPPLEMENTARY MATERIALS












