# Supplementary material for: FH535, a β-catenin pathway inhibitor, represses pancreatic cancer xenograft growth and angiogenesis
Source: Oncotarget. 2016 Jun 13;7(30):47145–62. doi: 10.18632/oncotarget.9975 (PMC5216931; doi:10.18632/oncotarget.9975)
Supplement: Supplementary file 1 [file oncotarget-07-47145-s001.pdf]

FH535, a β-catenin pathway inhibitor, represses pancreatic cancer xenograft growth and angiogenesis

Supplementary Materials

Inclusion and exclusion criteria of meta-analysis

The studies would be included in our meta-analysis if they met the following inclusion criteria: (1) β-catenin was examined by immunohistochemistry and evaluated in human pancreatic cancer tissues; (2) evaluation of the relationships between β-catenin expression and overall survival (OS) of pancreatic cancer; (3) English language publications; (4) sufficient information provided to estimate the hazard ratio (HR) and 95% confidence intervals (CIs). The following articles were excluded: (1) case reports, reviews, letters, and conference abstracts without original data or articles with no full text; (2) insufficient information provided to estimate the HR and its 95% CI; (3) articles from which the relevant data could not be extracted; (4) overlapping articles or those with duplicate data; (5) non-English language articles.

Statistical analysis of meta-analysis

All the data were independently extracted from the articles, tables, figures, and supplement of the publications by two authors (LW and WMY). The database recorded the most relevant data comprising author's name, year of publication, study location, number of patients, age of patients, antibody source, dilution, method, definition of abnormal β-catenin expression, and hazard ratio source. HR with 95% confidence interval (CI) was used to estimate the association between β-catenin expression and the OS of pancreatic cancer patients. For studies which had not given HRs directly, Kaplan-Meier curves were read by Engauge Digitizer version 4.1.

Heterogeneity across studies was evaluated using a Chi-square-based Q statistical test. The I2 statistic was also calculated to quantify the proportion of the total variation due to study heterogeneity. A $P > 0.05$ for the Q-test indicated a lack of heterogeneity among the studies. For studies with $P > 0.05$ the pooled HR estimates of each study were calculated by the fixed-effects model (the Mantel-Haenszel method). For studies with $P \leq 0.05$, the random-effects model (the DerSimonian and Laird method) was used. Funnel plot and Egger's test was used to examine the potential risk of publication bias. The statistical analyses were performed using STATA version 12.0 software (Stata Corporation, Collage Station, Texas, USA). All the P values were used for a two-sided test with significance at $P < 0.05$.

The criteria of evaluating β-catenin immunohistochemical staining

The intensity of staining was graded on a 4-point scale of 0, 1, 2 and 3 (with 3 as the most intense staining). The extent of positive immunoreactivity was graded by the percentage of stained cells in the region of interest: 0 points for 0%, 1 point for < 20%, 2 points for 20%~50% and 3 points for > 50%. An overall score was obtained by the product of intensity and extent of positive staining. Cases with 0 points were considered to be negative, cases with a final score of 1~3 as weakly positive, cases with a score of 4~7 as moderately positive and cases with a final score of more than 7 as strongly positive.

Angiogenesis vascularity evaluation

Angiogenesis vascularity was defined as the number of vessels per field counted in the area of highest vascular density, termed as microvessel density (MVD). Endothelial cells were marked with anti-CD34 antibody. CD34 antigen was localized in the cytoplasm and cellular membrane of vascular endothelial cells. Single endothelial cells, endothelial cell clusters, and microvessels in the tumors, clearly separated from adjacent microvessels, were counted. Peritumoral vascularity and vascularity in areas of necrosis were not scored. A vascular lumen was not a requirement for a structure to be counted as a microvessel. Branching structures were counted as one, unless there was a break in the continuity of the vessel, in which case it was counted as two distinct vessels. Areas with a higher density of CD34+ cells and cell clusters relative to adjacent areas were classified as 'hot spots'. The slides were initially screened at low power to identify the areas with the highest number of microvessels or vascularity hot spots. Microvessels were counted in 400× magnification fields. MVD was defined as the number of manually counted vessel profiles per mm^2 taken as the average from the three hot-spot counts.

GO (gene ontology) analysis

GO analysis was applied to analyze the main function of the differential expression genes according to the Gene Ontology which is the Wey functional classification of NCBI [13, 14]. Generally, Fisher's exact test and χ^2 test were used to classify the GO category, and the false discovery rate (FDR) was calculated to correct the P-value, the smaller the FDR, the small the error in judging

the P-value. The FDR was defined as $FDR = 1 - \frac{N_k}{T}$, where N_k refers to the number of Fisher's test P-values less than χ^2 test P-values. We computed P-values for the GOs of all the differential genes. Enrichment provides a measure of the significance of the function: as the enrichment increases, the corresponding function is more specific, which helps us to find those GOs with more concrete function description in the experiment. Within the significant category, the enrichment Re was given by: $Re = (n_f / n)/(N_f / N)$, where n_f is the number of differential genes within the particular category, n is the total number of genes within the same category, N_f is the number of differential genes in the entire microarray, and N is the total number of genes in the microarray.

Supplementary Table S1: GO-Analysis of microarray data. See Supplementary_Table_S1

Supplementary Table S2: Protein:DNA binding sites obtained from ChIP-Seq data by using peak calling. See Supplementary_Table_S2

Supplementary Table S3: Promoter specific binding sites obtained from ChIP-Seq data by using peak calling. See Supplementary_Table_S3

Supplementary Table S4: Motif analysis of ChIP-Seq data. See Supplementary_Table_S4

Supplementary Table S5: GO-Analysis of ChIP-Seq data. See Supplementary_Table_S5
